# Whole-Genome Analysis of *Escherichia coli* from One Health Sources: Evaluating Genetic Relatedness and Antimicrobial Resistance Carriage

**DOI:** 10.3390/antibiotics14111151

**Published:** 2025-11-14

**Authors:** Alyssa Butters, Juan Jovel, Sheryl Gow, Cheryl Waldner, Sylvia L. Checkley

**Affiliations:** 1 Faculty of Veterinary Medicine, University of Calgary, Calgary, AB T2N 4Z6, Canada; 2 AMR—One Health Consortium, Calgary, AB T2N 4Z6, Canada; 3 Canadian Integrated Program for Antimicrobial Resistance Surveillance, Public Health Agency of Canada, Saskatoon, SK S7N 5B4, Canada; 4 Department of Large Animal Clinical Sciences, Western College of Veterinary Medicine, University of Saskatchewan, Saskatoon, SK S7N 5B4, Canada

**Keywords:** antibiotic resistance, phylogenetics, comparative genomics, molecular epidemiology, *Escherichia coli*, One Health

## Abstract

**Background/Objectives**: Due to the numerical dominance of environmental and commensal strains, understanding antimicrobial resistance (AMR) transmission in *Escherichia coli* requires consideration of non-clinical as well as pathogenic isolates. In this cross-sectional study, associations between the genetic context of non-clinical *E. coli* and AMR carriage are examined in isolates sampled from different niches within a One Health continuum. **Methods**: Two hundred eighty-eight *E. coli* isolates collected in Alberta, Canada (2018–2019) from wastewater, well water, feces of broiler chickens and feedlot cattle, and retail beef and chicken meat were selected from existing surveillance collections using a stratified random sampling structure. Using short-read whole genome assemblies, phylogenetic relationships were inferred from pan-genome multiple sequence alignments. Principal coordinate analysis and permutational analysis of variance (PERMANOVA) of a Jaccard dissimilarity matrix derived from gene presence/absence data were used to investigate contributions of source and AMR strata to observe genetic dissimilarity. Population clustering and gene under- or over-representation by source and cluster were also explored. **Results**: Minimal phylogenetic segregation of isolates was noted based on source or AMR strata, and both contributed significant but small proportions of observed genetic dissimilarity, with the largest proportion attributed to phylogroup. There was notable diversity of *E. coli* within and between sources; however, in some larger clusters, differential gene presence/absence was potentially linked to ecological niche rather than source of isolation. **Conclusions**: This study highlights the ecological complexity of AMR in *E. coli* in non-clinical contexts, offering a novel lens on how niche-specific factors can influence population structure and AMR carriage. It also provides insight into apparent discrepancies in the literature regarding clustering of *E. coli* by source. These findings support a more integrative One Health approach to AMR surveillance, emphasizing the need to account for microbial diversity and niche-specific adaptation across interconnected systems.

## 1. Introduction

Antimicrobial resistance (AMR) is a critical health threat [1]; therefore, a thorough understanding of factors mediating its transmission is crucial to efforts to mitigate its impact and preserve life-saving antimicrobial therapies [2]. Selection for antimicrobial-resistant bacteria can arise from the use of antimicrobials in animal and plant agriculture or to treat human disease, and can be propagated by inadequate infection control or the international movement of humans and animals [3,4]. Humans, animals, and the environment have been highlighted as AMR reservoirs [3], emphasizing the need for a One Health approach to reducing the impact of AMR [1]. However, to date, our collective understanding of the bacterial acquisition and transmission of AMR in a One Health context remains fragmented and incomplete [5].

*Escherichia coli* is a model organism in the One Health study of AMR, as it can exist within most domains of the One Health continuum. Although a common commensal (naturally living in the intestines without causing disease) of both humans and animals, some *E. coli* strains are also capable of causing intestinal and extra-intestinal disease [6,7,8]. This bacterium can also be found in environmental samples, such as water and soil [9], which was initially considered to represent recent fecal contamination as the bacterium was thought to be incapable of extended survival in the environment. It is now noted that some *E. coli* strains can not only survive but also reproduce in environmental compartments [10,11], and are termed “naturalized” environmental strains. Although *E. coli* is ubiquitous within humans, animals, and the environment, the extent to which its genetic relatedness across One Health domains influences the carriage of antimicrobial resistance remains poorly understood.

In examining the potential for AMR transmission between One Health domains, some studies have proposed that *E. coli* from animal, human, and environmental sources could form genetically distinct clusters. Two studies [12,13] found genetic clustering of extended-spectrum β-lactamase (ESBL) producing *E. coli* isolated from distinct sources within a One Health continuum, speculating that the adaptation of isolates to their respective environments could limit the transfer of isolates and potentially AMR between niches. Naturalized strains in wastewater treatment plants [14,15], beef processing facilities [16], and other non-host sources [10,17] can be genetically distinct from host-adapted strains. Genetic barriers to gene transfer between naturalized environmental *E. coli* and enteric-adapted strains have been proposed [18]. It has also been identified that the genetic context, specifically phylogenetic group, can affect the antimicrobial resistance carried by *E. coli* [19,20]. These limited examinations, however, do not examine the full diversity of *E. coli* broadly within a One Health context.

Previous One Health studies addressing the influence of genetic context on AMR transmission in *E. coli* often focus on pathogenic strains or isolates carrying resistance to specific antimicrobials of high priority to human health [21]. These studies frequently rely on the retrospective analysis of publicly archived isolates, which can over-represent clinical pathogens isolated from diseased hosts or isolates with complex resistance elements [22]. This underrepresents the numerically more abundant, non-clinical *E. coli* that inhabit commensal and environmental niches [23]. This omission of non-clinical strains (from non-infectious contexts such as environmental samples or non-diseased hosts) is further complicated by non-random sampling, leading to disproportionate representation of specific subpopulations and obscuring comprehensive demographic associations [24]. Additionally, failure to define spatial and temporal boundaries in sampling could lead to unappreciated or potentially undefined variation and bias of isolates within and between sampling niches [5].

The objectives of this study were to determine the genetic relatedness of non-clinical *E. coli* isolated from production animal feces, related retail meat commodities, wastewater, and well water in isolates from samples collected in a single Canadian province in a limited time frame (2018–2019). In addition, it aimed to investigate if the genetic context of the isolates was associated with the carriage of AMR, as delineated using the unparalleled genetic discrimination afforded by whole-genome sequencing. Broadly, the intent was to acquire insight into the transmission of AMR between potential animal and environmental reservoirs, and whether differences in genetic background between *E. coli* strains in different One Health sources could influence AMR carriage. It was hypothesized that isolates from the same source would cluster genetically, and that genetic similarity would be associated with similarity in AMR carriage.

## 2. Results

### 2.1. Sequencing

Whole-genome short-read sequencing was completed on 288 *E. coli* isolates. One retail beef sample was determined to be a duplicate as it matched another isolate’s serotype, sequence type, identified antimicrobial resistance genes, and the pair had an average nucleotide identity of >99.99%. It was removed from further analysis. The remaining 287 samples were sequenced to a mean estimated sequencing depth of 197× (range 64.0–294.0×), down-sampled during assembly by Shovill to a mean final coverage of 79.52× (range 49.0×–116.0×) to reduce “noise” (sequencing errors) from excess sequencing data, which can diminish assembly quality once sufficient coverage has been reached (Supplementary Table S2). In the resultant assemblies, the mean genome length was 5.05 Mb (minimum 4.51 Mb, maximum 5.96 Mb), and the isolates demonstrated a GC content between 50.2% and 50.91%. The N_50_ value was >50,000 for all isolates.

### 2.2. Phylogenetic Analysis

**Figure 1:**
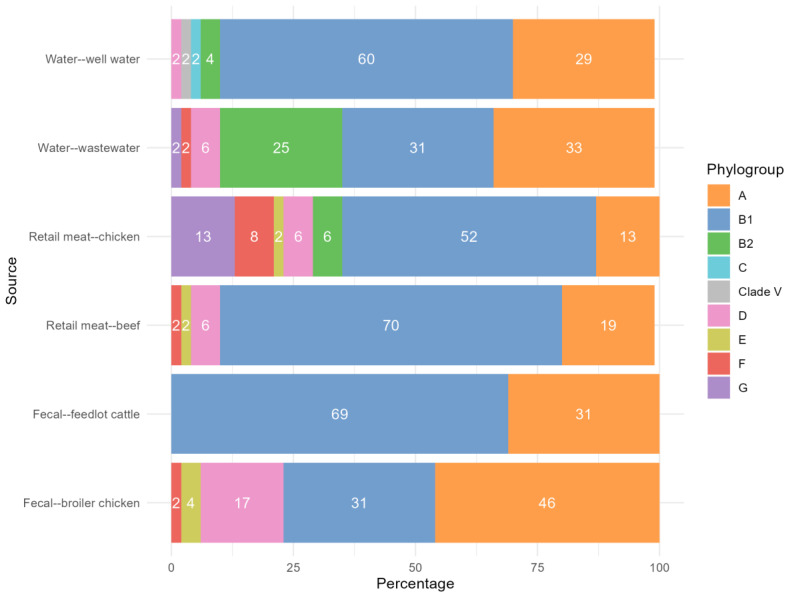
Distribution of phylogroups represented by *E. coli* isolates from feedlot feces, broiler chicken feces, retail beef, retail chicken meat, wastewater, and well water, as a percentage of the total for each source.

Phylogroups B1 and A were over-represented among isolates from all sources, totaling 52.3% and 28.6% of all isolates sequenced. Phylogroup B1 was the most numerous phylogroup of isolates in feedlot fecal, retail beef, retail chicken, and well water samples; Phylogroup A was the most common phylogroup in sequenced isolates from wastewater and broiler chicken fecal samples (Figure 1). Phylogroup D isolates were most common in *E. coli* from broiler chicken feces (*n* = 8, 16.7%), and represented 6% of isolates sequenced from wastewater, retail chicken meat, and retail beef. Phylogroup G was only identified in retail chicken meat (*n* = 6, 12.5%) and wastewater (*n* = 1, 2.1%), and phylogroup B2 was most frequent among wastewater isolates (*n* = 12, 25%), followed by isolates from retail chicken meat (*n* = 3, 6.3%) and well water (*n* = 2, 4.2%). There were 148 unique sequence types (STs) represented within the samples, although 11 isolates did not have an ST assigned (Table 1, Supplementary Table S3). The most common ST was ST10, present in 23 samples and found in all sources. It was the most frequently identified ST among feedlot cattle and broiler chicken fecal samples. Other common STs included ST58 (13 isolates, found in feedlot fecal, retail beef, retail chicken, and well water samples), ST155 (10 isolates, found in all sources), and ST399 (9 isolates from retail beef, wastewater, and well water).

**Figure 2:**
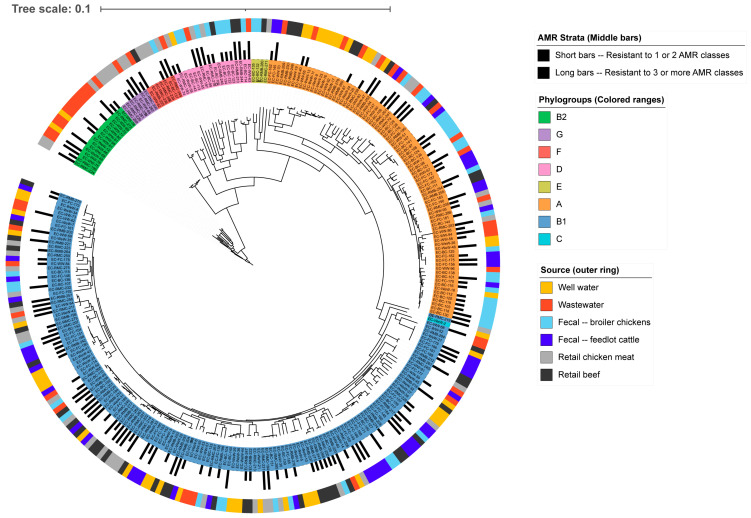
Pan-genome maximum likelihood phylogenetic tree of *E. coli* isolates. The tree is rooted at B2 isolates.

All isolates clustered by phylogroup on the phylogenetic tree, except for a single phylogroup C isolate that was grouped with B1 (Figure 2). No clear clustering by source was observed, apart from a grouping of isolates from retail chicken meat and a single wastewater isolate belonging to phylogroup G. Similarly, isolates showed minimal segregation by AMR strata. However, a small cluster of pan-susceptible isolates, primarily from well water, was observed within phylogroup A, a source notably overrepresented among pan-susceptible isolates.

**Figure 3:**
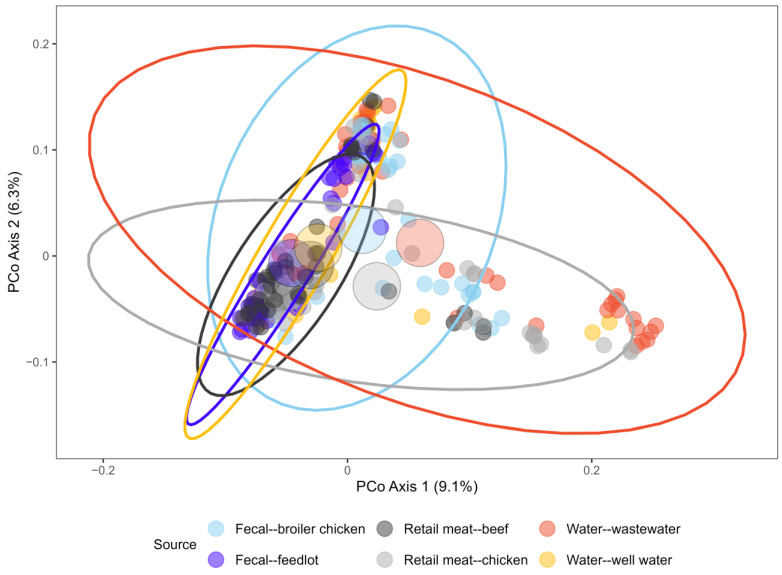
Principal coordinate analysis of *E. coli* isolates by isolation source, based on a Jaccard distance matrix (pangenome gene presence/absence). Larger coloured points represent centroids of the distribution for each source, and ellipsoids represent a 95% confidence interval surrounding the centroid of each source.

**Figure 4:**
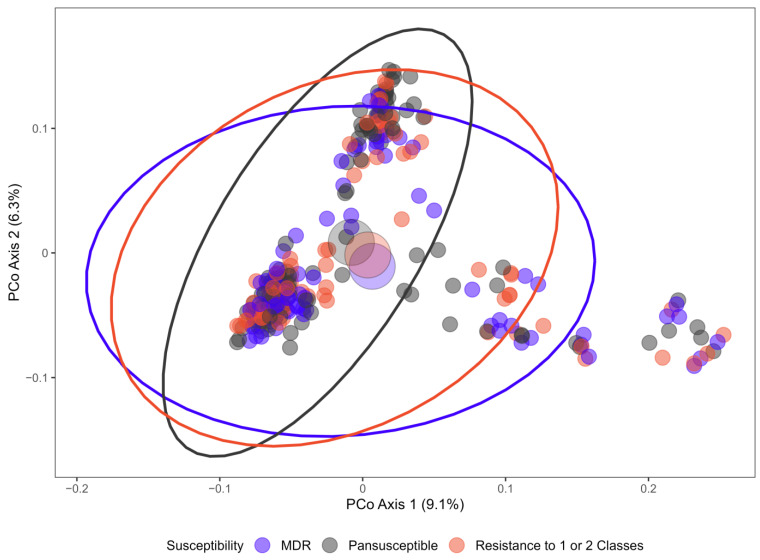
Principal coordinate analysis of *E. coli* isolates by AMR strata based on a Jaccard distance matrix (pangenome gene presence/absence). Larger coloured points represent centroids of the distribution for each AMR strata, and ellipsoids represent a 95% confidence interval surrounding the centroid of each AMR strata. MDR = multidrug resistance (resistance to 3 or more classes).

Genetic similarity of the isolates was also assessed through analysis of gene content. Pan-genome analysis identified a total of 22,256 genes, of which 3054 were found to be core genes (present in ≥99% of isolates). PERMANOVA analysis indicated that the centroid and/or spread of the ordination points demarcating isolates from different sources (Figure 3) and AMR strata (pan susceptible, resistant to 1 or 2 antimicrobial classes, multidrug resistant) (Figure 4) were statistically different (*p* = 0.001 for both). Although this identified that gene presence and absence patterns vary among isolates from different sources and AMR strata, these factors represented only small portions of the explained variance in the data (R^2^ = 0.047 and R^2^ = 0.012, respectively). Phylogroup accounted for the largest portion of the explained variance (R^2^ = 0.239, *p* = 0.001), and the interaction between phylogroup and source also represented a statistically significant but small portion of the explained variance (R^2^ = 0.035, *p* = 0.001).

There was heterogeneity of dispersion between groups (the group variances differed) for each of phylogroup, source, and AMR strata, which threatens the underlying assumption of PERMANOVA that the observations are exchangeable under the null hypothesis. However, PERMANOVA is relatively robust to heterogeneity if the sampling design is balanced, a condition met for the “source” variable, with very similar numbers of isolates sampled for each source (*n* = 47–48). In contrast, the number of isolates within each phylogroup is inherently unbalanced within the dataset, due to the unequal representation of the phylogroups among the sampled isolates and the over-representation of some phylogroups. However, the ordination plot for phylogroup (Supplementary Figure S1 demonstrates clear separation of the centroids and ellipses for each phylogroup, suggesting a centroid difference rather than a dispersion effect).

Given the unbalanced sampling design with regard to AMR strata, with a predominance of pan-susceptible isolates among well water isolates, the PERMANOVA results could be influenced by differences in dispersion rather than centroid differences. The largest group (pan-susceptible well water isolates) had lower dispersion, which can bias the PERMANOVA pseudo-F statistic in a liberal direction, increasing the likelihood of detecting spurious group differences. Considering this, the PERMANOVA analysis was also performed with all well water isolates removed, which equalized the number of samples in each AMR strata. Similar results were obtained in this subsequent analysis, with significant but small portions of the variance explained by source (R^2^ = 0.044, *p* = 0.001), AMR stratum (R^2^ = 0.015, *p* = 0.001), and the interaction between source and AMR stratum (R^2^ = 0.031, *p* = 0.001). As previously, the largest proportion of the explained variance was represented by phylogroup (R^2^ = 0.283, *p* = 0.001). Given the minimal impact on results following the removal of well water isolates, the influence of the unbalanced AMR strata sampling was likely negligible.

The impact of the Clade V outlier was also evaluated. When this isolate was excluded but the well-water isolates retained, PERMANOVA results remained unchanged, except for a very small shift in the R^2^ values for the phylogroup (R^2^ = 0.238, *p* = 0.001 without the Clade V isolate vs. R^2^ = 0.239, *p* = 0.001 with the Clade V isolate). The Clade V outlier was not influential.

### 2.3. Gene Presence/Absence by Source

Assessing gene over- and under-representation by source, as determined using Scoary, was completed to identify source-specific selective pressures or ecological adaptations that shape the functional potential of *E. coli* populations across different One Health environments. Associations between gene presence/absence and source of sampling did not identify any statistically over- or under-represented genes in isolates from broiler fecal, retail beef, wastewater, or well water samples (Supplementary Table S4). Twenty-six genes were over-represented in isolates from retail chicken, including nine annotated by Prokka as “hypothetical proteins.” Other over-represented genes in this source are implicated in iron homeostasis (*fes_2*, *iucA*, *iutA*), iron/manganese transport (*mntB_1*&*2*), and other export/efflux systems (fluoride: *crcB_2*; toxin and drug export: *tdeA*; macrolide antibiotics: *macA_2*; multi-drug efflux: group_20002).

Within isolates from feedlot fecal samples, 61 over-represented genes and two under-represented genes were found. The under-represented genes from this source were *mntB_1* and *mntB_2*, which are involved in iron/manganese transport and were otherwise over-represented within retail chicken meat *E. coli* isolates. Most over-represented genes in *E. coli* from feedlot cattle feces encoded hypothetical proteins (*n* = 38). Genes in the hlyABCD operon, a type I secretion system responsible for exporting the hemolysin toxin, were also over-represented in feedlot fecal *E. coli* isolates, especially among isolates from phylogroup B1. Genes involved in arginine biosynthesis or metabolism (*argF*, *argR*, *arcA_2*) were over-represented in *E. coli* isolates from feedlot cattle fecal samples. Also over-represented in feedlot fecal isolates were genes encoding toxins used in defense against other bacteria (colicin A, encoded by *cia_1*/group_3011; *cidA*, a part of a toxin-immunity protein module involved in a cellular contact-dependent growth inhibition system) and transposase genes from the IS110 and IS3 families.

### 2.4. Cluster Analysis

In the whole dataset, variable-length *k*-mer [25] clustering by PopPUNK was used to define clusters of isolates that exhibit significant similarity in both core and accessory genome sequence and gene content, relative to the rest of the isolates [26]. This identified notable diversity within the isolates, delineating 135 unique clusters, most comprising four or fewer isolates (Supplementary Table S3). Only nine clusters contained five or more isolates, with the largest cluster (Cluster 11) containing 28 isolates. Isolates of the same ST were grouped into the same cluster with some exceptions [e.g., isolates from ST10 were found in Clusters 5 (*n* = 14), 28_753 (*n* = 5), 1408 (*n* = 2), 979 (*n* = 1), and 92 (*n* = 1)]. Clusters were generally not defined by source, as most larger clusters contained isolates from several sources. For example, isolates from all six sources were found in Cluster 11 (Table 2). However, some clusters (e.g., Cluster 21) comprised isolates mostly from a single source (retail chicken meat *n* = 6, wastewater *n* = 1; Table 2).

**Figure 5:**
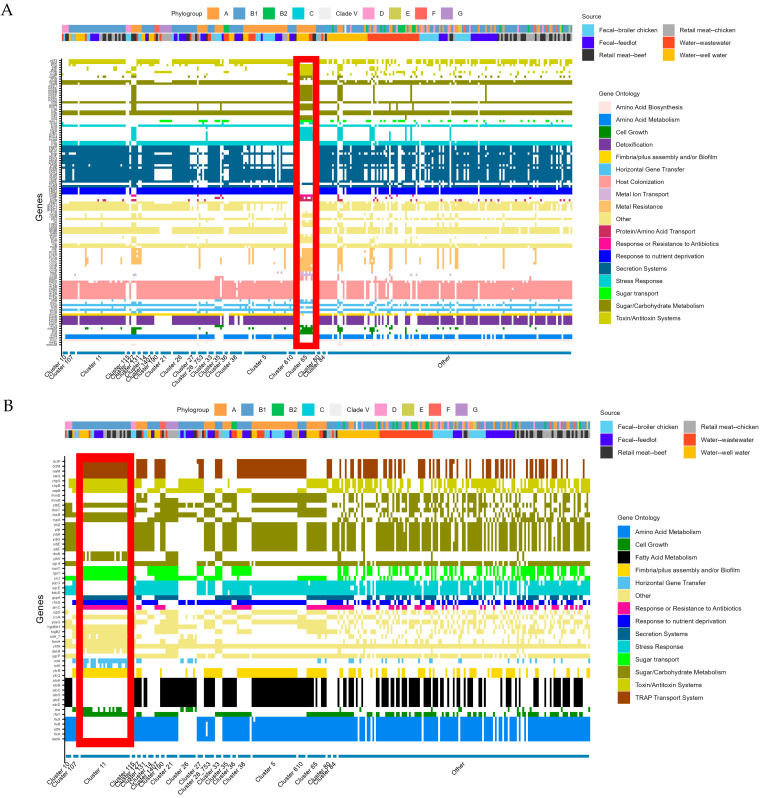

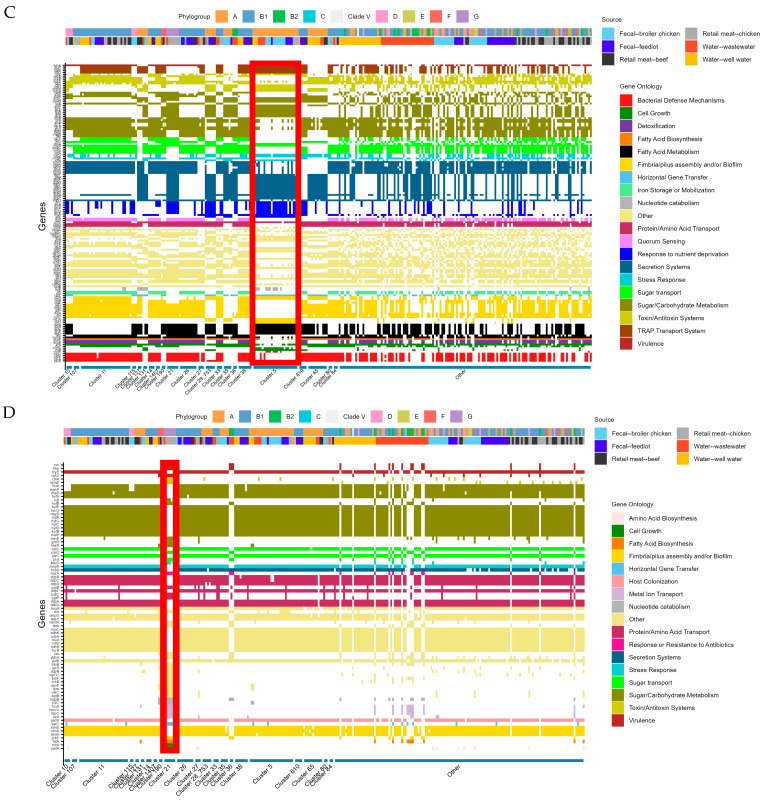
Heatmap of gene over- and under-representation, phylogroup, and isolation source among *E. coli* isolates from (**A**). Cluster 65, (**B**). Cluster 11, (**C**). Cluster 5, (**D**). Cluster 21, compared to all other isolates from the dataset. In each pane, isolates from the cluster of interest are identified with a red box.

To identify genes or gene functions that could define each cluster and its unique conditions, gene over- and under-representation was assessed statistically using Scoary, following the same approach used for source-based comparisons. This analysis revealed statistically significant gene representation patterns in only four clusters: Clusters 5, 11, 21, and 65, comprising 25, 28, 7, and 12 isolates, respectively. Functional analysis of over- and under-represented genes in each cluster (Figure 5A–D) showed distinct profiles of gene enrichment and depletion, particularly in pathways related to sugar and carbohydrate metabolism, toxin–antitoxin systems, and virulence.

Isolates in Cluster 65 demonstrated differential gene presence/absence in genes related to stress response, metal ion transport, and metal resistance (Figure 5A). Several genes involved in secretion systems (mainly type II secretion systems), host colonization, and response to nutrient deprivation were notably under-represented among Cluster 65 isolates. The cluster comprised *E. coli* from well water, wastewater, and retail beef of ST399 and ST635 belonging to Phylogroup A, and all Cluster 65 isolates were susceptible to all tested antimicrobials, except for one wastewater isolate phenotypically resistant to nalidixic acid.

Cluster 11 exhibited a gene representation pattern distinct from that of Cluster 65, with several under-represented genes associated with amino acid and fatty acid metabolism and stress response (Figure 5B), alongside over-represented genes involved in sugar transport and other metabolic functions. Isolates from Cluster 11 belong to Phylogroup B1 and several different STs (ST58, ST155, ST616, ST683, and ST5565), representing isolates from all sources examined in this dataset. In terms of AMR carriage, all well water isolates in this cluster (*n* = 5), two from retail chicken, and one from retail beef were susceptible to all antimicrobials tested. All other isolates in the cluster demonstrated phenotypic resistance to at least one antimicrobial tested.

Conversely, Cluster 5 demonstrated under-representation of genes related to bacterial defense mechanisms, sugar transport, and fimbria/pilus assembly and/or biofilm formation (Figure 5C). Genes related to fatty acid metabolism and response to nutrient deprivation were also over-represented among isolates in Cluster 5. This cluster contained a diversity of STs and sources of sampling, containing *E. coli* isolated from all sources except retail beef and isolates from several different STs (ST10, ST43, ST744, ST752, ST1141, ST5265, ST6617, and non-typable). This diversity was also demonstrated in AMR carriage by isolates within this cluster, with each source represented in this cluster (except for feedlot fecal samples) contributing both resistant and susceptible isolates. All feedlot fecal samples in this cluster were resistant to at least one antimicrobial tested.

Cluster 21, a cluster of seven phylogroup G isolates of ST117 from retail chicken meat and wastewater, was the least diverse in STs represented among the four clusters with over- or under-represented genes. The cluster had several genes related to metal ion transport over-represented among its isolates (Figure 5D). Regarding AMR carriage, two isolates (both from retail chicken meat) were susceptible to all antimicrobials tested; the remainder of the isolates in this cluster were resistant to at least one antimicrobial tested.

Chi-squared tests were used to determine whether any cluster exhibited a statistically different rate of AMR carriage. Cluster 11 demonstrated a significant over-representation of isolates resistant to three or more antimicrobial classes (χ^2^ = 7.48, df = 2, *p* = 0.024). However, this over-representation did not achieve statistical significance when applying the Bonferroni-adjusted significance level (0.0125). No other cluster showed an association with antimicrobial AMR carriage at frequencies significantly different from those expected within the stratified random sampling framework (1/3 of isolates susceptible, 1/3 of isolates resistant to 1 or 2 antimicrobial classes, 1/3 of isolates resistant to 3 or more classes).

## 3. Discussion

This study leverages the unparalleled genetic discrimination of whole-genome sequencing to examine the genomic landscape of numerically abundant commensal and environmental *E. coli* from various facets of a One Health continuum. Furthermore, it examines the associations between the carriage of AMR and the genetic similarity/dissimilarity of the geographic and temporally constrained isolates selected by a stratified random sampling approach.

The *E. coli* isolates from food animal production and water sources were diverse, with only 3054 of 22,256 identified genes found in ≥99% of isolates. The genetic relatedness of non-clinical *E. coli*, as determined by phylogenetic pan genome analysis, demonstrated minimal segregation of isolates by source or antimicrobial resistance. These two variables contributed only minimally to the total explained variance in genetic dissimilarity among isolates, as indicated by significant but small R^2^ values (<0.05) from PERMANOVA based on a Jaccard distance matrix derived from gene presence/absence data. This limited explanatory power is visually evident in the ordination plots, where group centroids are relatively close together and the 95% confidence ellipses overlap substantially. Minor effects should be interpreted with caution, as these can reach statistical significance due to large sample sizes or the complexity of the data and model structure. Despite noted heterogeneity in the dispersion of the isolates, neither the unbalanced sampling design (over-representation of susceptible isolates among *E. coli* from well water) nor the Clade V outlier appeared to be influential, as results differed minimally after removal of these isolates.

These findings of substantial diversity and minimal segregation by source or AMR are consistent with other recent studies that also did not find associations between *E. coli* isolates and host species [21,27,28,29], country [28], geographic sublocation [27], or genetic markers of AMR [28]. The striking diversity of isolates in this study echoes the findings of other examinations of non-clinical *E. coli* isolates [21,29].

In contrast, some previous studies have identified genetic segregation of isolates from different One Health sources [12,13]. Discrepancies between their findings and this study could stem from differences in isolate types, resistance profiles, and geographic proximity of sampling. Ludden et al. [12] identified a genetic segregation of *E. coli* isolates based on the source of sampling (livestock or humans), but their analysis specifically compared clinical *E. coli* isolates from human bloodstream infections with non-clinical and ESBL-producing *E. coli* isolates from livestock. Clinical isolates could represent a more selective subset of the *E. coli* population, where clustering is more likely due to the narrower selective pressures of host–pathogen interactions and healthcare-associated factors, such as antimicrobial use. Similarly, focusing on the ESBL phenotype rather than the general *E. coli* population could also have increased the likelihood of detecting related strains in sources affected by runoff from confined cattle feeding operations, compared to human-associated isolates, in the study by Adator et al. [13]. Additionally, that study sampled cattle feedlots, adjacent catch basins, and surrounding streams in immediate proximity to compare the genetic similarity of *E. coli* isolates related to beef cattle production, then compared to isolates from beef processing plants, municipal sewage, and human clinical specimens that were linked geographically but not as closely as the beef cattle production isolates in that study.

Muloi et al. [27] noted some genetic similarity among *E. coli* isolates from individual households that was not detected when *E. coli* from different households were compared, again indicating that the geospatial scale can affect the degree of genetic similarity identified. Supporting this, Shaw et al. [29] noted a lack of association between *E. coli* sequence type and individual livestock species, although farm-specific distinctions were observed and persisted over time. It therefore seems plausible that finding genetic similarity of *E. coli* isolates from sampling sites in very close proximity could be reconciled with a more “cosmopolitan distribution” [27] when extending this sampling more broadly, resolving the apparent disparity in findings from studies demonstrating genetic clustering of *E. coli* isolates in some contexts and the lack of such clustering in others.

Genetic segregation by source was assessed by identifying genes that were over- or under-represented among sources using gene presence/absence data analyzed with Scoary, following the developers’ recommendation for exploratory, non-causal association. This was pursued to identify functional manifestations of potential source-specific ecological adaptations. However, few over- or under-represented genes or gene functions were identified in most sources, indicating that source-specific variations in gene content were only detected infrequently. This aligns with findings from Touchon et al. [21], where *E. coli* from most sources showed limited enrichment of specific genes or functions except isolates from freshwater, in which distinct patterns of gene family over- and under-representation were observed. Additionally, genes found to be over- or under-represented in the current study’s dataset could reflect functional adaptations to specific ecological niches, rather than being uniquely associated with the source. Among isolates from feedlot cattle feces, many over-represented genes are not unique to the host species. Instead, these genes enhance the survival of *E. coli* in highly competitive microbial environments typical of a commensal existence, allowing efficient nitrogen utilization (*argF*, *argR*; [30]), optimized and flexible switching between aerobic and anaerobic metabolism (*arcA2*; [31]), and defense against other bacteria (colicin A, *cidA*; [32]), or have been found commonly in B1 phylogroup isolates from other mammals in addition to cattle [33]. The significance of under-representation of *mntB_1* and *mntB_2* (implicated in manganese transport) within *E. coli* from feedlot cattle feces is less clear.

A similar lack of specificity to the source of isolation was also seen within over-represented genes in isolates from retail chicken *E. coli*. Virulence genes *ompT* and *iutA,* and genes related to metal and iron acquisition systems, such as aerobactin (*iucA* in this dataset) [25,34], were notably over-represented among isolates from retail chicken meat but not chicken origin isolates in general, as the genes were less common in isolates from broiler chicken feces. However, several of these genes (e.g., *iutA*, *iucA*) can mediate environmental stress responses [25] and could represent niche-specific selection to broad ecological conditions. During poultry processing, isolates bearing APEC virulence genes conferring increased survivability in harsh and often nutrient-limited meat processing environments could be at a selective advantage [35]. This leads to the selection of isolates carrying genes that confer a selective advantage under conditions present within, but not exclusive to, a particular source or host species.

Because of the observed lack of genetic or functional segregation by source, variable-length *k*-mer clustering by PopPUNK was completed to identify groups of isolates, unconstrained by source delineations, that demonstrate similarity in sequence and gene content. This approach again revealed notable diversity in the sampled *E. coli*, with 135 unique clusters defined. Consistent with the earlier observation of minimal source-associated genetic structure, most of these clusters contained isolates from multiple sources. In contrast to the limited number of genes differentially represented within sources, several of these *k*-mer–partitioned clusters exhibited distinct patterns of gene enrichment or absence relative to other *E. coli* clusters in the dataset.

Taken together, the absence of source-specific genetic segregation, finding minimal over- or under-represented genes by source, and the identification of genetic (*k*-mer) clusters containing isolates from several sources highlight that the concept of “ecotypes” might be a more useful construct when assessing *E. coli* populations. Ecotypes are genetic subpopulations of a given bacterial species that fill a defined ecological niche [36]. One such potential ecotype observed in this dataset is the cluster of phylogroup A isolates of ST399 and ST635 identified in well water, wastewater, and retail beef (Cluster 65), two sequence types previously identified as naturalized inhabitants in manufactured environments involving water sanitation or meat processing [14,15,16]. Like the naturalized isolates in Yu et al. (2024) [15], *E. coli* in Cluster 65 demonstrated over-representation of genes involved in stress response and under-representation of genes encoding secretion systems, metal acquisition, and colonization factors. Cluster 65 isolates also displayed under-representation of genes involved in response to nutrient deprivation, consistent with environmental *E. coli* that generally do not partition energy to nutrient scavenging under nutrient-limited conditions, exhibiting a so-called “survival-like phenotype” [9]. These gene patterns support the assertion that these isolates represent “naturalized” *E. coli* in this environment rather than contaminants of fecal origin.

The increased frequency of isolation of ST117 from retail chicken meat and wastewater isolates could also be consistent with niche selection of bacterial subpopulations. There is emerging evidence that the avian pathogenic *E. coli* (APEC) lineage ST117 *E. coli*, which in this dataset all belonged to Cluster 21 (*n* = 6 from retail chicken, *n* = 1 from wastewater), has been associated with human extraintestinal infections such as urinary tract infections, bloodstream infections, and pneumonia [37,38]. It can also colonize the human gut [37,38]. ST117 has most commonly been identified in chicken meat [37], as noted herein. The source of most extraintestinal pathogenic (ExPEC) isolates contaminating retail chicken meat is thought to be the gastrointestinal tract of processed carcasses [35]; however, in studies identifying ST117 in chicken feces, the reported prevalences were relatively low, ranging from 0.4% [39] to 5.6% [40] and 6.6% [41] In this dataset, six ST117 isolates were found in retail chicken meat, but none were found in broiler chicken isolates, despite sampling using the same stratified random selection. Environmental factors (high temperature, disinfecting agents) in the meat processing environment could select for the persistence of these APEC *E. coli*. The genetic factors allowing this persistence could also be responsible for increased virulence in humans [25], an important consideration worthy of further research for mitigating foodborne infections.

The finding of relatively few under- or over-represented genes by source also supports the concept that ecotype rather than source could be a more relevant construct in considering the genetic relatedness of bacterial sub-populations. Genetically similar isolates within an ecotype could originate from multiple sources, thereby minimizing the number of genes that are statistically over- or under-represented in any given source. Consistent with this, PopPUNK clusters (isolates identified as statistically similar in shared sequence and gene content in the core and accessory genomes) are more alike in sequence type than in host origin or AMR carriage, as noted herein and by others [28]. The presence of more “generalist” strains or clusters able to exist in multiple hosts or environmental locations, like Cluster 11, containing isolates from all sources in this study, would similarly obscure source-specific gene presence/absence patterns. Some investigators [42] have commented that it should not be surprising to find minimal genetic clustering of isolates by host, given the typical concomitant presence of both generalist and host-specialized commensal *E. coli* strains within most host species. These generalist strains, present in multiple sources, could also potentially mediate some genetic exchange between more specialist ecotypes [43]. This could be a plausible mechanism for AMR transmission between ecotypes, but further research is required to confirm or refute this hypothesis.

In addition to shaping ecotypes, environmental selection forces, including temperature, pH, nutrient availability, solar radiation, salinity, and heavy-metal presence, can influence the survivability of *E. coli* in the environment [9,44] and could also conceivably affect the acquisition and maintenance of antimicrobial resistance genes by environmental bacteria. Interestingly, other studies have identified that freshwater isolates might also carry antimicrobial resistance at lower frequencies [21,45], consistent with the low resistance levels among well water isolates in this examination. The influence of the well water environment on AMR carriage was also suggested by the patterns of resistance seen within an additional cluster (Cluster 11), chiefly comprised isolates of ST58 and ST155. All well water isolates in Cluster 11 were susceptible to all antimicrobials tested, whereas most isolates from other sources in this cluster (except three from retail meats) demonstrated resistance to at least one antimicrobial. Although not statistically significant, the higher multidrug resistance in Cluster 11 isolates from non-well water sources warrants further investigation into the selective influence of the water environment on AMR carriage. Others have noted the trend for antimicrobial resistance carriage by *E. coli* belonging to ST58 and ST155 [46]. Skurnik et al. [46] did not observe a difference in the AMR carriage of isolates from these STs based on sampling origin, but their study included only commensal *E. coli* from humans and animals and no environmental isolates. Overall, little is understood about how environmental factors could influence the potential transmission of AMR, indicating an area that merits additional study.

Well water isolates from this dataset concur with other findings that “naturalized” *E. coli* populations represent a distinct, well-adapted population in freshwater environments [47], shaped by the environmental factors defining this niche. Strains from the B1 phylogroup have demonstrated prolonged persistence in freshwater [45], consistent with the predominance of B1 isolates among well water *E. coli* in this study. Tropea et al. [48] also found phylogroup B1 isolates were the most frequently isolated *E. coli* from private well water and further contend that approximately 30% of wells had an “entirely adapted community” of *E. coli* based on a dimensionally reduced *E. coli* adaptation model. Further, Walk et al. [47] hypothesized that isolates from freshwater beaches represented a relatively stable environmental *E. coli* population independent of site, based on similar diversity estimates, biotypes, genotypes, and phylogroup distribution between sites and minimal between-site contribution to genetic diversity of the isolates. The phylogroup proportions they reported are comparable with those seen within well water isolates within the current study. Cookson et al. [49] additionally identified that recent fecal inputs to freshwater systems were associated with a higher diversity of *E. coli* phylotypes, not dominated by phylotype B1 isolates as was seen in *E. coli* freshwater populations with what was described as “non-recent, aged contamination events.” Together, these findings support the growing body of evidence contesting the paradigm that environmental *E. coli* isolates must necessarily be derived from recent fecal contamination events [21] and instead seem to represent a unique subpopulation shaped by the selective influences particular to this environment.

The frequent isolation of certain sequence types from distinct niches (ST399/ST635 from water and food processing-associated environments, ST117 from processed chicken fecal at higher frequency than chicken feces) suggests that selective conditions unique to a given niche could result in subpopulations or ecotypes uniquely well-adapted to a microenvironment that is not limited to a particular animal species or environmental milieu. This could also be consistent with the clustering reported in household- or farm-level sampling that is not seen when sampling a broader geospatial scale [27,29], as individual farms or households could have unique selective pressures [29], that are inconsistent when considering different locations, even from the same livestock commodity or geographic region.

The authors acknowledge some considerations constraining the conclusions drawn from this study. The prescribed framework for stratified random sampling could not be fulfilled for well water isolates due to the overrepresentation of susceptible isolates from that source. Despite this, the proportional paucity of phenotypic AMR in these well water isolates was notable. However, this study cannot determine whether these results stem from selection pressure on resistant isolates to shed AMR elements, from differential survival of susceptible and resistant isolates, or whether *E. coli* inputs to the well water system carried lower resistance levels. Also, the initial identification screening process (Colilert^®^) completed on the well water isolates will not detect O157:H7 *E. coli* isolates, as O157:H7 lacks β-glucuronidase activity [50] and therefore will not metabolize the 4-methylumbelliferyl-β-D-glucuronide hydrate (MUG) present in the Colilert system to produce a fluorescent product, as will other *E. coli* strains. This prevents the extension of the study findings to O157:H7 isolates. However, the impact of this omission is anticipated to be small, as previous estimates of O157:H7 prevalence in private drinking water wells within Alberta have been very low (0.1%) [51].

While this study’s approach does not directly assess the mobility or context of specific AMR genes, it provides foundational insight into the potential for cross-source transmission of *E. coli* and the AMR these bacteria carry. A logical next step in understanding AMR dissemination would involve detailed characterization of AMR gene content of these isolates and the mobile genetic elements that mediate their transfer, potentially with the additional genetic discrimination provided by long-read sequencing.

It would be illuminating to examine how antimicrobial use (AMU) is associated with AMR carriage by *E. coli* within each source of sampling. However, relevant AMU data were not available for the samples studied and integrating AMU estimates from broader populations with AMR data from the individual samples in this study would introduce bias. The addition of relevant, appropriate AMU data in future One Health research examining AMR transmission would be a valuable addition to the current literature. Also, due to financial and logistical constraints, sampling within this study was limited to two production animal commodities and two water sources. While this represents only a small snapshot of the microbial diversity within a One Health continuum, the study offers a unique perspective on the intersection of genetic relatedness and AMR carriage among *E. coli* isolates, without limitation to specific AMR profiles or pathotypes. Furthermore, it considers the potential role of non-clinical commensal and environmental *E. coli* in the broader transmission dynamics of AMR.

## 4. Materials and Methods

### 4.1. Sample Collection

*Escherichia coli* was isolated from feedlot cattle feces, broiler chicken feces, retail beef, retail chicken meat, post-treatment wastewater, and private well water samples collected in Alberta, Canada, in 2018 and 2019. Comprehensive methodologies for bacterial collection and isolation have been described previously [52].

Briefly, *E. coli* isolates from retail meat and fecal samples were obtained from routine surveillance sampling by the Canadian Integrated Program for Antimicrobial Resistance Surveillance (CIPARS). For surveillance of fecal bacteria, farms participate voluntarily in the program and were selected to be representative of farm locations and sizes found in Canada for each commodity. Veterinary consulting clinics providing regular veterinary services to the farm collected the samples. For broiler chickens, 4 pooled fecal samples (at least 10 fecal droppings per sample) from the barn floor were collected according to a defined random sampling grid in the final week preharvest (>30 days of age). For each participating feedlot, pooled pen-floor fecal samples were collected from 10 pens with cattle close to market weight. Fecal samples were shipped to the laboratory on ice.

For retail meat sampling, CIPARS purchased ground beef (very lean, lean, medium, and regular) and skin-on chicken legs or wings directly from retail establishments and shipped them in zipper-type plastic bags on ice to the laboratory. Stores were selected using a stratified random sampling method defined by Statistics Canada census data and weighted by province/region. Four Alberta stores were typically sampled each collection week: 3 chain grocery stores and one butcher shop or independent market.

From 5 January 2018 to 8 November 2019, weekly wastewater sampling after final UV treatment (directly before release from the treatment facility) was completed at three wastewater treatment plants in Calgary, Alberta, Canada. Detailed descriptions of sample collection procedures and wastewater treatment plant characteristics have been published previously [53].

Well water samples submitted to Alberta Precision Laboratories as part of drinking water testing between 19 July 2018, and 19 December 2019, were included in the study. Samples were collected voluntarily at the discretion of private citizens. Homeowners collected untreated water from residential cold-water supply lines and submitted it on ice within 24 h. Kits were obtained from provincial public health clinics, along with detailed instructions for collection.

### 4.2. Bacterial Isolation, Identification, and Phenotypic Antimicrobial Susceptibility Testing

Detailed methodologies for bacterial isolation from CIPARS fecal and retail meat samples have been previously described [54]. In short, 25 g of pooled cattle or chicken feces was mixed with 225 mL of buffered peptone water (BPW), then one drop of the resultant mixture was streaked onto MacConkey agar and incubated at 35 ± 1 °C for 18–24 h. Lactose-fermenting colonies were assessed for purity, plated on Luria–Bertani (LB) agar, and incubated at 35 ± 1 °C for 18–24 h before confirmation of identity with Simmon’s citrate and indole testing. Any indole-negative isolates were subjected to additional testing for identity with the API^®^ 20E Identification System following the manufacturer’s specifications (bioMerieux, Marcy-L’Etoile, France). Most fecal samples were processed at the National Microbiology Laboratory (NML) in Saint-Hyacinthe, Quebec, with a few undergoing primary isolation at Alberta Agriculture and Irrigation’s Agri-Food Assurance Laboratory.

Retail meat samples (1 chicken leg or wing, or 25 g of ground beef) were mixed with 225 mL of BPW. Fifty millilitres of the mixture were combined with an equal amount of double-strength EC broth and incubated at 42 ± 1 °C for 24 h, then one loopful was streaked onto Eosin Methylene Blue (EMB) selective agar. EMB plates were then incubated at 35 ± 1 °C for 18–24 h. One well-isolated, metallic green colony was selected from each plate, streaked onto trypticase soy agar with 5% sheep’s blood, and incubated at 35 ± 1 °C for 18–24 h before identity testing was completed as for fecal isolates. Retail meat samples were processed at the NML.

At the Water Quality Services Laboratory (UEP—Water Resources, City of Calgary), each wastewater sample was filtered through a 0.45 µm Buchner filter, which was then placed on m-FC agar and incubated at 44.5 °C overnight. At the University of Calgary, blue colonies (coliforms) were sterilely picked and added to a single well of a 96-well plate, each of which contained 100 µL of sterile Luria–Bertani (LB) broth. After overnight incubation, sterile LB broth with glycerol was added to each well to achieve a final concentration of 25% glycerol. Plates were frozen at −80 °C. One post-treatment coliform isolate, thus cryopreserved, was randomly selected from each available wastewater sampling date at each treatment plant for further identification and antimicrobial susceptibility testing (192 isolates).

Well water samples were tested at Alberta Precision Laboratories with a commercial enzymatic substrate test kit (Colilert^®^, Idexx Laboratories, Westbrook, ME, USA) for coliforms and *E. coli* presence. Those demonstrating a yellow colouration and fluorescence (presumptive *E. coli* positive samples) had a small aliquot (1 mL) retained and cryopreserved at −80 °C with or without 15% glycerol before being transferred to the University of Calgary (*n* = 88).

Cryopreserved well water samples were thawed on ice, and 1 mL of the sample was added to 9 mL of sterile tryptic soy broth, then incubated at 37 °C for 18–24 h. For cryopreserved wastewater samples, isolates were revived in 10 mL of sterile tryptic soy broth (TSB) and incubated similarly. Ten microlitres of each overnight incubated TSB + well water or TSB + wastewater sample was plated onto X-Gluc agar and re-incubated at 37 °C for 18–24 h. One random, well-isolated blue colony (presumptive *E. coli*) was selected from each X-Gluc plate and sterilely streaked onto tryptic soy agar with 5% sheep’s blood. After 18–24 h incubation at 37 °C, the bacterial identity of the sample was confirmed using the API^®^ 20E Identification System.

All samples were subjected to phenotypic antimicrobial susceptibility testing via automated broth microdilution (Sensititre^TM^ Trek Diagnostic Systems Ltd., West Sussex, UK) at either the NML (isolates from retail meat and fecal samples) or the University of Calgary (wastewater and well water). National Antimicrobial Resistance Monitoring System (NARMS) Gram-negative CMV4AGNF plates were used, testing for susceptibility to amoxicillin–clavulanic acid, ampicillin, azithromycin, cefoxitin, ceftriaxone, chloramphenicol, ciprofloxacin, gentamicin, meropenem, nalidixic acid, streptomycin, sulfisoxazole, tetracycline, and trimethoprim–sulfamethoxazole. *Escherichia coli* ATCC 25922, *Pseudomonas aeruginosa* ATCC 27853, *Staphylococcus aureus* ATCC 29213 and *Enterococcus faecalis* ATCC 29212 were used as quality control strains. Clinical and Laboratory Standards Institute (CLSI) breakpoints [55] were used to interpret susceptibility results to all antimicrobials tested except azithromycin and streptomycin, for which CLSI interpretation criteria are not available for Enterobacterales. For azithromycin and streptomycin, NARMS breakpoints were used (https://www.fda.gov/media/108180/download?attachment; last accessed on 24 October 2025).

### 4.3. Whole Genome Sequencing

A total of 288 *E. coli* isolates were selected for whole genome sequencing according to a stratified random sampling based on source and number of class-level resistance phenotypes demonstrated. Forty-eight isolates from each source were selected: 16 isolates susceptible to all antimicrobials tested, 16 isolates with phenotypic resistance to 1 or 2 classes of antimicrobials, and 16 isolates demonstrating phenotypic resistance to 3 or more classes of antimicrobials. Isolates assigned intermediate antimicrobial susceptibility were categorized with susceptible isolates. An insufficient number of well-water isolates showed resistance to meet these sampling criteria. Therefore, all phenotypically resistant well water isolates (*n* = 4) were included, and 44 non-resistant *E. coli* were randomly selected from the remaining well water isolates. Class level resistance of isolates from each One Health source is detailed in Supplementary Figure S2 and Supplementary Table S1. All random sampling utilized the sample_n() function in the base package of R v 4.3.2 using RStudio 2024.09.1 (build +394) [56,57] with a seed set using a random number generator.

DNA was extracted from the selected *E. coli* isolates using DNeasy Blood and Tissue kits (Qiagen, Toronto, ON, Canada). After DNA quantification with a Qubit Fluorometer 3.0 (Thermo Fisher Scientific, Mississauga, ON, Canada) and sample purity assessment by spectrophotometry (NanoVue Plus; GE HealthCare, Chicago, IL, USA), the samples were submitted to the Centre for Health Genomics and Informatics at the University of Calgary (Calgary, AB, Canada) for short-read whole-genome sequencing. RIPTIDE^®^ High-Throughput Rapid Library Prep (iGenomX/Twist Bioscience, Carlsbad, CA, USA) was used for library preparation for most isolates, followed by sequencing on the Illumina NovaSeq 6000 platform (San Diego, CA, USA) to produce 2 × 250 base pair paired-end reads. However, a small number (*n* = 7) of isolates were re-sequenced from original stocks with an Illumina MiSeq v2 (San Diego, CA, USA) after library prep with the NEBNext Ultra II (New England BioLabs Inc., Ipswich, MA, USA) to produce paired-end reads of the same length, due to contamination (*n* = 1) or mislabelling of the original sequencing samples (*n* = 6). The re-sequenced assemblies replaced the original assemblies in all analyses.

### 4.4. Bioinformatics

Raw reads were assessed using fastqc v 0.11.9 [58], then trimmed with fastq-mcf v 1.04.807 [59] with the following parameters: skew percentage of 10 (k 10), minimum length of 120 (−l 120), sliding window of three (−w3), and a minimum Phred quality score of 20 (−q 20). Reads were assembled de novo using SPAdes (v 3.13.1) [60] within the Shovill pipeline (DNA) using default settings, and the resultant assemblies were quality-assessed using Quast v 5.2.0 [61] and checked for completeness and contamination with CheckM v 1.1.6 [62]. Sequence type and phylogroup were assigned in silico using mlst v 2.23.0 (https://github.com/tseemann/mlst, accessed on 24 October 2025, which makes use of the PubMLST database [63]), and ClermonTyping v 21.03 [64], respectively. When assigned phylogroup and Mash group did not match (e.g., ClermonTyping note: “Phylogroup doesn’t match the mash closest neighbor’s group! This could indicate a mutation affecting the binding of a primer”), the Mash group designation was used, as Mash groups reflect overall genome similarity rather than reliance on a limited set of loci [64] and Mash group designation is better suited to capturing broad evolutionary relationships. Assemblies were annotated with Prokka v 1.14.6 [65] with the “--species Escherichia” flag.

Pan-genome analysis was completed with Panaroo v 1.3.3 [66] using the mafft aligner v 7.407 [67] with the flags “a pan” (to output alignments of all genes rather than core genes only) and “--clean_mode strict” (retaining only genes present in at least 5% of genomes to minimize potential sources of contamination and error). The pan genome multiple sequence alignment produced by Panaroo formed the input for phylogenetic tree inference with iqtree v 2.2.0.3 [68] with a GTR + F + I + I + R10 model selected using ModelFinder [69] and ultra-fast bootstrapping completed with UFBoot2 [70]. The tree was visualized and annotated with metadata using Interactive Tree of Life (iTOL), v 6.9; [71], rooted at B2 isolates consistent with the proposed evolutionary history of *E. coli* phylogroups [19,72]. Average nucleotide identity of isolates was compared using FastANI v 1.34 [73] and population analysis and clustering was assessed with PopPUNK v 2.6.3 [26].

After sequencing, all isolates for which the AMR phenotype did not match the AMR genes assigned by ResFinder/Pointfinder [74,75,76] were retested phenotypically for AMR using the broth microdilution method outlined above. One well water isolate originally determined to be phenotypically resistant to streptomycin was susceptible to all antimicrobials on retesting, and was reclassified as pan susceptible, consistent with its identified genotype. It is suspected that this discrepancy could have resulted from either contamination of the initial broth microdilution inoculum or loss of a plasmid during subsequent culturing for cryopreservation and sequencing.

### 4.5. Statistical Analysis

The significance level was set at *p* < 0.05 for interpretation of all statistical tests unless otherwise noted.

A Jaccard dissimilarity matrix was calculated in RStudio [56,57] with the micropan package v 2.1 [77] from the pangenome gene presence/absence data derived by Panaroo. This was carried out with and without the single Clade V isolate, which represented a distinct visual outlier. Permutational multivariate analysis of variance (PERMANOVA) on the Jaccard dissimilarity matrix was completed using the adonis2 function within the vegan R package v 2.6-4 [78] with 999 permutations. For the PERMANOVA, phylogroup, source, and AMR strata (pan susceptible, multi-drug resistant, or resistant to 1 or 2 antimicrobial classes) were entered as factors, and an interaction between phylogroup and source was included. The multivariate homogeneity of group dispersions (i.e., the variability of samples around their respective group centroids) was tested using the betadisper() function from the vegan R package. Statistical significance of the dispersion differences was evaluated via permutation testing using permutest(), also from vegan. Together, these functions test whether the spread of samples within groups differs significantly across groups. Unequal dispersions can violate the PERMANOVA assumption of exchangeability under the null hypothesis. When group dispersions are large, between-group differences may be artificially inflated relative to within-group variation, potentially leading to statistically significant PERMANOVA R^2^ values even when group centroids do not differ.

Principal coordinate analysis (PCoA) based on the Jaccard dissimilarity matrix was also carried out using the vegan package. The PCoA results were visualized using ggplot2 v 3.4.5 from the tidyverse R package v 2.0.0 [79], with 95% confidence intervals demarcated as ellipses around the centroids for each stratum.

Pan-genome associations between gene presence or absence (ascribed by Panaroo) and isolate source were determined using Scoary v 1.6.16 [80]. As the objective of this analysis was to identify genes enriched within each sampling source rather than to infer causal associations, the “--no_pairwise” flag was applied in accordance with the developers’ recommendations. For each gene, Scoary calculated an odds ratio indicating the strength of association between the presence of the gene and a particular isolate source. If the odds ratio for gene presence was greater than 1 for a given source, it indicated that the gene was more frequently present in isolates from that source compared to all other sources and was therefore designated as over-represented in that source. Conversely, if the odds ratio was less than 1, the gene was present less frequently in isolates from that source relative to others and was considered under-represented. In these calculations, a Benjamini–Hochberg corrected *p*-value cut-off of 1 × 10^−5^ was used as the statistical threshold of significance. Functional annotations for genes thus identified were assigned based on gene ontology descriptions within the Uniprot database [81].

A Pearson’s χ^2^ test or Fisher’s Exact test (used when expected values were <5) was employed to evaluate associations between resistance strata and PopPUNK cluster. This analysis was completed in RStudio using the stats package v 4.3.2 for all clusters in which over- or under-represented genes were identified (*n* = 4; Clusters 5, 11, 21, and 65). Well water isolates were excluded from this analysis due to an over-representation of pan-susceptible isolates in that source because of the insufficient number of resistant isolates available to meet the stratified random sampling criteria. For each assessed cluster, a binary variable was created to indicate cluster assignment (1 = assigned to cluster, 0 = not assigned to cluster). A 2 × 3 contingency table was constructed for each cluster, comparing cluster assignment to resistance strata (susceptible, resistant to 1–2 classes, or resistant to ≥3 classes). Based on the stratified random sampling approach, the expected distribution for any given cluster was one-third of isolates in each resistance category. The χ^2^ or Fisher’s Exact test was used to assess whether the observed distribution within each cluster significantly deviated from this expectation. A Bonferroni correction was applied to the significance threshold to account for multiple comparisons in over- and under-represented genes among clusters (κ = 4, adjusted *p* = 0.0125).

## 5. Conclusions

*Escherichia coli* isolates from well water, wastewater, broiler chicken and feedlot cattle feces, and retail beef and chicken meat demonstrated marked diversity, with little genetic segregation by source or carriage of phenotypic AMR. Despite this, some clustering of isolates was observed, corresponding to ecological niches rather than the source of isolation. The presence of ecotypes that span sources could reconcile the observed absence of gene over- and under-representation among isolates from most sampling sources. The relative scarcity of AMR among well water isolates and the trend toward higher prevalence of AMR carriage among ST58 and ST155 isolates suggest that niche differentiation and certain environmental or niche-specific factors could influence AMR carriage. The finding of genetically similar *E. coli* across multiple sources suggests that source alone is unlikely to act as a barrier to AMR dissemination. Collectively, the findings of this study would seem consistent with the ecological tenet that “everything is [almost] everywhere, but the environment selects.”

Therefore, when evaluating if *E. coli* from a given source could represent a reservoir or conduit for transmission of AMR genes or bacteria, the suite of environmental factors that shape the population composition could also influence AMR transmission dynamics and should be additional considerations beyond the presence of *E. coli* in each site or host species. At a local scale, *E. coli* isolates could be particularly well-adapted to specific micro-environments, resulting in clustering patterns that are not apparent at broader geographic or ecological scales. These distinctions warrant careful attention in both the design of sampling methodology for future surveillance programs and the interpretation of data from One Health studies. As ecological niches can span multiple sampling sources, integration of niche-specific environmental factors and population structures will improve AMR model predictions. This perspective supports a shift in surveillance and mitigation strategies from targeting broad sources (e.g., host species or environments) to focusing on specific ecological niches that may act as reservoirs or conduits for AMR transmission. Such an approach could enhance the precision of One Health risk assessments and improve the effectiveness of AMR interventions.

## Figures and Tables

**Table 1:** *E. coli* sequence types by source of isolation.

Source	Sequence Types	Number of Isolates
Broiler chicken fecal	10	6
	189	3
	720	3
	752	3
	155	2
	2705	2
	328	2
	40	2
	Singletons	25
Feedlot cattle fecal	10	7
	1080	3
	10,895	3
	6488	3
	-	2
	155	2
	20	2
	278	2
	336	2
	58	2
	6617	2
	Singletons	18
Retail chicken meat	117	6
	58	4
	10	3
	295	3
	-	2
	101	2
	602	2
	648	2
	Singletons	24
Retail beef	58	4
	109	3
	155	3
	607	3
	10	2
	399	2
	56	2
	847	2
	Singletons	26
Wastewater	-	4
	10	3
	998	3
	Singletons	38
Well water	399	6
	1086	3
	58	3
	10	2
	442	2
	635	2
	Singletons	30

**Table 2:** Source, phylogroup, sequence type, and level of antimicrobial resistance carriage of *E. coli* assigned to PopPUNK clusters that demonstrated statistically over- or under-represented genes.

Cluster	Number of Isolates	Sources (*n*)	Phylo-Group	Sequence Types (*n*)	AMR Strata (*n*)
Cluster 65	12	Water—well water (8)	A	399 (9)	Pan susceptible (11)
		Water—Wastewater (2)		635 (3)	Resistant to 1 or 2 classes (1)
		Retail meat—beef (2)			
Cluster 11	28	Water—well water (5)		58 (13)	Pan susceptible (8)
		Water—wastewater (1)	B1	155 (10)	Resistant to 1 or 2 classes (7)
		Fecal—broiler chicken (2)		616 (1)	Resistant to 3 or more classes (13)
		Fecal—feedlot cattle (5)		683 (1)	
		Retail meat—chicken (7)		5565 (1)	
		Retail meat—beef (8)		Not typed (2)	
Cluster 5	25	Water—well water (3)	A	10 (14)	Pan susceptible (7)
		Water—wastewater (4)		43 (2)	Resistant to 1 or 2 classes (11)
		Fecal—broiler chicken (10)		744 (1)	Resistant to 3 or more classes (7)
		Fecal—feedlot (5)		752 (3)	
		Retail meat—chicken (3)		1141 (1)	
				5265 (1)	
				6617 (2)	
				Not typed (1)	
Cluster 21	7	Water—wastewater (1)	G	117 (7)	Pan susceptible (2)
		Retail meat—chicken (6)			Resistant to 1 or 2 classes (2)
					Resistant to 3 or more classes (3)

## Data Availability

The original data (sequencing reads) presented in the study are openly available in the National Center for Biotechnology Information (NCBI) Sequence Read Archive under BioProject PRJNA1120594. Accessions can be found in Table S2 (SAMN41704486–SAMN41704772).

## Supplementary Materials

The following supporting information can be downloaded at: https://www.mdpi.com/article/10.3390/antibiotics14111151/s1, Figure S1: Principal coordinate analysis of *E. coli* isolates by AMR strata based on a Jaccard distance matrix (pangenome gene presence/absence). Figure S2: Class level antimicrobial resistance demonstrated by *E. coli*, before stratified random sampling; Table S1. Number of isolates and phenotypic antimicrobial resistance demonstrated by *E. coli* from feedlot and broiler chicken fecal samples, retail beef and chicken meats, post-treatment wastewater, and well water sampled in Alberta, Canada 2018–2019 prior to stratified random sampling; Table S2: Summary of sequencing depth, coverage, contig numbers, and assembly quality metrics for *E. coli* short-read genome assemblies; Table S3: Variable length *k*-mer clusters of *E. coli* genomes based on short-read whole-genome sequencing, as assigned by PopPUNK; Table S4: Over and underrepresented genes among *E. coli* isolates of each isolation source, as assigned by Scoary.

## Abbreviations

The following abbreviations are used in this manuscript:

AMR	Antimicrobial resistance
APEC	Avian pathogenic *Escherichia coli*
CIPARS	Canadian Integrated Program for Antimicrobial Resistance Surveillance
CLSI	Clinical and Laboratory Standards Institute
EMB	Eosin-methylene blue
ExPEC	Extraintestinal pathogenic *Escherichia coli*
LB	Luria–Bertani
Mb	Megabasepairs (1,000,000 base pairs)
NARMS	National Antimicrobial Resistance Monitoring Systems (USA)
NML	National Microbiology Lab
PCoA	Principal Component Analysis
PERMANOVA	Permutational analysis of variance
ST	Sequence type
TSB	Tryptic soy broth
