# Supplementary material for: Whole-Genome Analysis of Escherichia coli from One Health Sources: Evaluating Genetic Relatedness and Antimicrobial Resistance Carriage
Source: Antibiotics (Basel). 2025 Nov 14;14(11):1151. doi: 10.3390/antibiotics14111151 (PMC12649636; doi:10.3390/antibiotics14111151)
Supplement: Supplementary file 1 [file antibiotics-14-01151-s001.zip › SUPPLEMENTARY MATERIAL Table S1 and Figures S1 and S2.pdf]

SUPPLEMENTARY MATERIAL

Table S1. Number of isolates and phenotypic antimicrobial resistance demonstrated by *E. coli* from feedlot and broiler chicken fecal samples, retail beef and chicken meats, post-treatment wastewater, and well water sampled in Alberta, Canada 2018-2019 prior to stratified random sampling

Source	Total number of isolates	Isolates non-resistant to all antimicrobials tested	Isolates resistant to one or two antimicrobial classes tested	Isolates resistant to three or more antimicrobial classes tested
Feces[a]				
Feedlot cattle	412 (100%)	181 (43.93%)	171 (41.50%)	60 (14.56%)
Broiler chickens	237 (100%)	86 (36.28%)	88 (37.13%)	63 (26.58%)
Retail Meats[a]				
Beef	225 (100%)	185 (82.22%)	24 (10.67%)	16 (7.11%)
Chicken	243 (100%)	98 (40.33%)	68 (27.98%)	77 (31.69%)
Wastewater[b]	116 (100%)	76 (65.52%)	22 (18.97%)	18 (15.52%)
Well water[c]	84 (100%)	81 (96.43%)†	1 (1.19%)†	2 (2.38%)

[a] Samples provided by the Canadian Integrated Program for Antimicrobial Resistance Surveillance, Public Health Agency of Canada

[b] Samples provided by Advancing Canadian Water Assets (Dr. Tao Dong's laboratory, University of Calgary) and the City of Calgary

[c] Samples provided by Alberta Precision Laboratories

†One isolate initially identified as resistant to streptomycin only was reclassified as susceptible based on whole-genome sequencing and repeat phenotypic testing

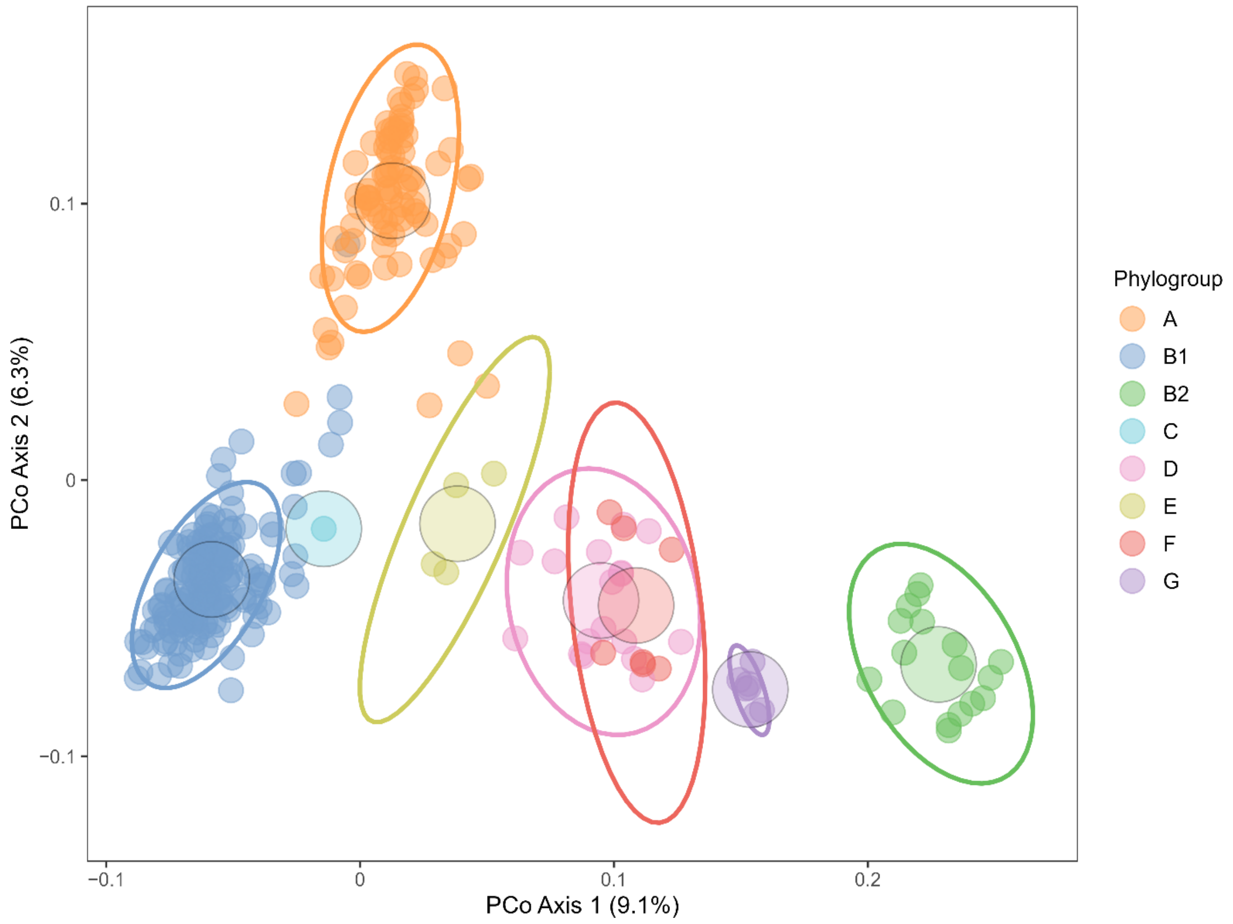

Figure S1. Principal coordinate analysis of *E. coli* isolates by phylogroup based on a Jaccard distance matrix (pangenome gene presence/absence). Larger coloured points represent centroids of the distribution for each phylogroup, and ellipsoids represent a 95% confidence interval surrounding the centroid of each phylogroup.

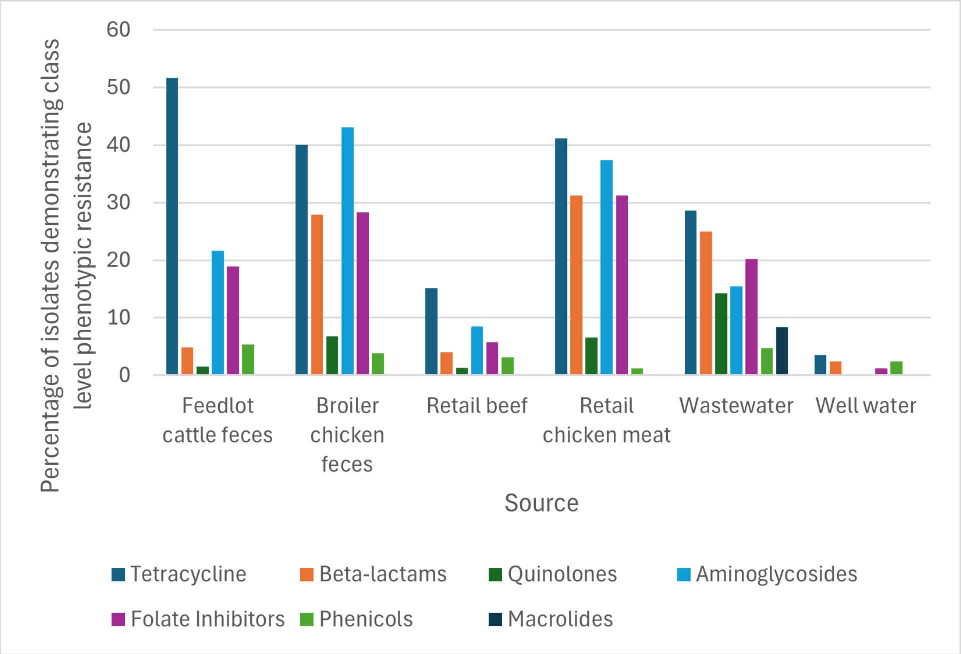

Figure S2. Class level antimicrobial resistance demonstrated by *E. coli* isolated from feedlot and broiler chicken fecal samples, retail beef and chicken meats, post-treatment wastewater, and well water sampled in Alberta, Canada, 2018-2019, before stratified random sampling.
